# Theoretical insights into the effect of halogenated substituent on the electronic structure and spectroscopic properties of the favipiravir tautomeric forms and its implications for the treatment of COVID-19

**DOI:** 10.1039/d1ra06309j

**Published:** 2021-11-01

**Authors:** Letícia Cristina Assis, Alexandre Alves de Castro, João Paulo Almirão de Jesus, Elaine Fontes Ferreira da Cunha, Eugenie Nepovimova, Ondrej Krejcar, Kamil Kuca, Teodorico Castro Ramalho, Felipe de Almeida La Porta

**Affiliations:** Department of Chemistry, Federal University of Lavras CEP 37200-000 Lavras Minas Gerais Brazil kamil.kuca@uhk.cz felipelaporta@utfpr.edu.br; Post-graduation Program in Materials Science and Engineering and Laboratory of Nanotechnology and Computational Chemistry, Federal Technological University of Paraná Avenida dos Pioneiros 3131 86036-370 Londrina Paraná Brazil; Department of Chemistry, Faculty of Science, University of Hradec Kralove Hradec Kralove Czech Republic; Center for Basic and Applied Research, Faculty of Informatics and Management, University of Hradec Kralove Hradec Kralove Czech Republic

## Abstract

In this study, we systematically investigated the electronic structure, spectroscopic (nuclear magnetic resonance, infrared, Raman, electron ionization mass spectrometry, UV-Vis, circular dichroism, and emission) properties, and tautomerism of halogenated favipiravir compounds (fluorine, chlorine, and bromine) from a computational perspective. Additionally, the effects of hydration on the proton transfer mechanism of the tautomeric forms of the halogenated favipiravir compounds are discussed. Our results suggest that spectroscopic properties allow for the elucidation of such tautomeric forms. As is well-known, the favipiravir compound has excellent antiviral properties and hence was recently tested for the treatment of new coronavirus (SARS-CoV-2). Through *in silico* modeling, in the current study, we evaluate the role of such tautomeric forms in order to consider the effect of drug-metabolism in the inhibition process of the main protease (M^pro^) and RNA-dependent RNA polymerase (RdRp) of SARS-CoV-2 virus. According to the molecular docking, all halogenated compounds presented a better interaction energy than the co-crystallized active ligand (−3.5 kcal mol^−1^) in the viral RdRp, in both wild-type (−6.3 to −6.5 kcal mol^−1^) and variant (−5.4 to −5.6 kcal mol^−1^) models. The variant analyzed for RdRp (Y176C) decreases the affinity of the keto form of the compounds in the active site, and prevented the ligands from interacting with RNA. These findings clearly indicated that all these compounds are promising as drug candidates for this molecular target.

## Abstract 2

In this study, we systematically investigated the electronic structure, spectroscopic properties, and tautomerism of halogenated favipiravir compounds (fluorine, chlorine, and bromine) from a computational perspective.

## Graphical abstract

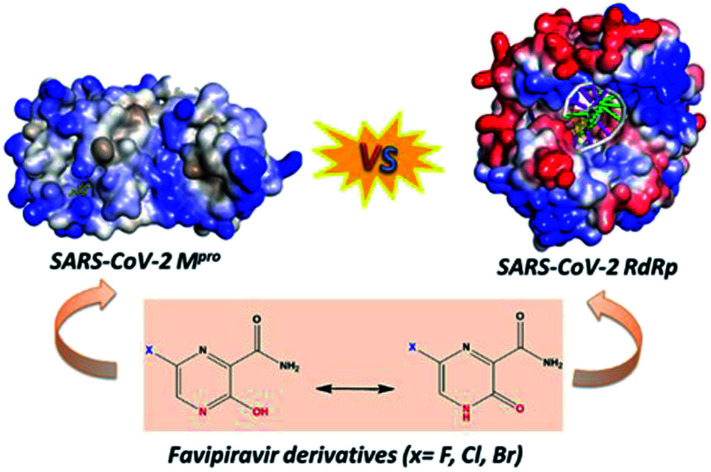

## 1. Introduction

Considerable efforts have been performed in a short period of time in search of therapeutic options for treatment of infection caused by a new coronavirus – SARS-CoV-2, which is the cause of the disease called COVID-19.^1–5^ Thus, researchers from all over the world have adopted as a strategy for treating COVID-19 infection attempting to inhibit two different types of known structural and non-structural proteins of the SARS-CoV-2 virus.^6–10^ As such, the first case involves the inhibition of the main protease (abbreviated as M^pro^) proteins of SARS-CoV-2.^9,11–14^ Notably, this structural M^pro^ protein displays a pivotal role because it is responsible for the cleavage of polyproteins. During this process, functional viral proteins and key enzymes for virus replication are released.^9,11,15^ In contrast, after the SARS-CoV-2 virus enters into the host cell, it is well-known that the RNA-dependent RNA polymerase (abbreviated as RdRp), a non-structural protein, is the main enzyme for the process of SARS-CoV-2 replication.^16–19^ Due to the eminent urgency to fight this COVID-19 outbreak, researchers around the world have widely evaluated the effectiveness of diverse approved antiviral agents for this purpose.^1–3,20–22^

**Fig. 1:**
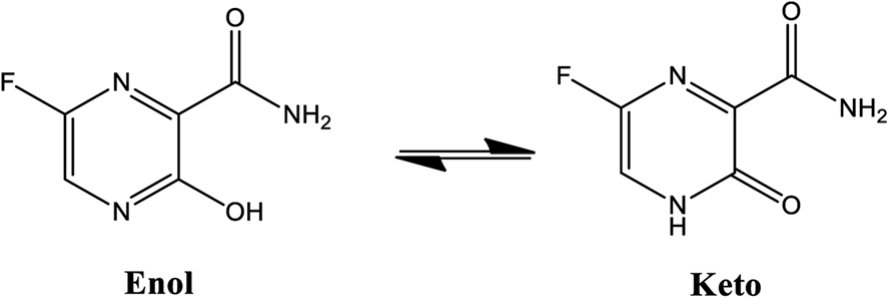
Chemical structure and tautomeric form of favipiravir.

Among these approved drugs, in particular, favipiravir (also known as T-705) is a compound analogous to guanine, which was developed with satisfactory activity against many RNA-polymerase viruses (*e.g.*, Ebola, chikungunya, yellow fever, influenza, norovirus and enterovirus),^23–28^ showed good clinical efficacy against coronavirus.^29,30^ Although the highly mobile protons in the structure of favipiravir compounds has allowed for their tautomeric forms,^31,32^ the interpretation of the spectroscopic properties of the tautomers has proven to be highly complex and difficult.^33^ Their chemical structure and tautomeric form are shown in Fig. 1.

Ongoing studies have focused on the understanding of the tautomeric forms of these compounds that are critically important for elucidating the nature of their chemical molecular behaviour.^31,33^ In contrast, quantum chemistry methods allow for the theoretical modelling of these spectral characteristics (*i.e.* to visualize this process on an atomic scale) quantitatively, which can, in principle, provide excellent opportunities for the design of new drugs.^33–37^ Some works dedicated to the study of favipiravir tautomerism, showing the importance of this topic. In this context, Antonov (2020) have reported from the density functional theory (DFT) based simulations that the enol form of favipiravir is preferred in both gas and solution phases.^32^ In other work, Yunusa *et al.* (2020) reported the rotational isomers, tautomeric states, electronic and spectral properties of favipiravir and its five analogues (Cl, Br, H, CN and CH_3_).^38^ They came to the conclusion that the aromatic enol tautomer is energetically more stable than the keto–enol tautomer in both gas phase and aqueous medium. However, there is a shift from the tautomeric equilibrium to the keto form in the aqueous phase, leading to a significant reduction in the relative energy of the keto–enol. This likely is due to the fact of the water environment has stabilized the more polar keto tautomer.^38^

Alver *et al.* (2019) investigated the adsorption between favipiravir and non-doped or silicon-doped C20 fullerenes, in order to investigate a possible application of the systems as drug delivery vehicles.^39^ These authors noticed that all systems have a large dipole moment, being this an essential criterion for drug interaction.^39^

Celik *et al.* (2021) investigated antiviral prodrugs such as favipiravir, remdesivir, galidesivir and ribavirin, and their triphosphate metabolites, for the viral RdRp inhibition.^40^ According to molecular docking results, triphosphate active metabolite forms showed higher interaction than prodrug and other intermediate metabolites.^40^ Sada *et al.* (2020) performed *in silico* studies to elucidate the molecular interactions of favipiravir with the viral RdRp of SARS-CoV-2, SARS-CoV, MERS-CoV and influenza virus.^41^ In conclusion, these authors found that favipiravir can bind to active sites of coronavirus RdRp proteins and replicated RNA terminals.^41^ In the same line, other *in silico* studies dedicated efforts in the study of drugs for COVID-19 treatment, such as favipiravir, remdesivir, among others.^42,43^

In this study, we focused on elucidating the effect of halogen (fluorine, chlorine, and bromine) on the electronic structure, spectroscopic (nuclear magnetic resonance, infrared, Raman, electron ionization mass spectrometry, UV-Vis, circular dichroism and emission) properties, and tautomerism of the favipiravir compounds from a computational perspective. In addition, since solvation has been known to play an important role in the tautomeric equilibrium,^31,33–36^ the solvent effect was considered in the transition state calculations for the tautomerism of the isolated, mono-hydrate, di-hydrate and tri-hydrated forms of the different halogenated favipiravir compounds. Herein, we also used *in silico* modelling for predicting the possible effects of drug-metabolism in terms of action and toxicity for the halogenated favipiravir compounds against the SARS-CoV-2 using both M^pro^ and RdRp sites as model systems.

For the accomplishment of this work, a range of computational techniques were employed. The theoretical tools were developed, among others, for the study of complex chemical and biological systems. The emergence of quantum mechanics (QM) to study molecules at electronic level was a remarkable achievement. The growth of methods based on classical physics to study large systems at the molecular level (MM) is also of great importance in the study of large systems, such as biomacromolecules. The parallel development of molecular simulations, which connects the macroscopic and the microscopic world elucidating the dynamical properties of molecules may guide the design of active potent molecules as therapeutic agents (computer-aided molecular design). In this context, we highlight the impact of theoretical chemistry on the advancement of our comprehension of complex chemical and biological systems. Thus, the comprehension of chemical and biological systems has reached greater heights due to an excellent harmony between experiment and theory.^44^ In order to illustrate the potential of theoretical and computational studies, we cite the study from Pierrefixe *et al.* (2008), which showed how and why carbon can become truly hypervalent under certain conditions.^45^

There are many positive consequences of these computational developments, particularly for treating systems in a more accurate fashion, and they can yield new insights. This study marks the first phase of a theoretical/experimental investigation of our group directed toward this goal. We hope that our results will stimulate new experimental and full-dimensional theoretical investigations that could assess the validity of this assumption.

## 2. Methods: computational details

Here, all quantum-chemical calculations were done through Gaussian 09 package.^46^ Full optimization and their frequencies of halogenated favipiravir compounds (fluorine, chlorine, and bromine) were achieved with Density Functional Theory (DFT) method at the B3LYP/6-31+G(d,p) level. Then, in order to consider the effect of drug metabolism, the transition states (TS) for the tautomeric forms of title compounds were computed through DFT calculations, at the same level of theory described previously. Additionally, the solvent effect (water) was considered in the TS calculations for the tautomerism of the isolated, mono-hydrate, di-hydrate and tri-hydrated forms using the polarizable continuum model (PCM).^47,48^ In addition, NMR calculations (in the gas phase and solution) were also performed for tautomeric forms of the halogenated favipiravir compounds at the B3LYP/6-31+G(d,p) level what do method.^49–53^ Time-dependent DFT (TD-DFT) calculations were also evaluated to obtain the UV-Vis, Electronic Circular Dichroism (ECD) and emission spectra, as well as their excitonic transitions, Molecular Orbitals (MOs) and Electrostatic Surface Potential (ESP) maps. Additionally, the Electron Ionization Mass Spectrometry (EI-MS) fragmentation spectrum for tautomeric forms and the trajectories of intermediaries were evaluated through semiempirical GFN2-xTB method as implemented in Quantum Chemistry Electron Ionization Mass Spectrometry program (QCEIMS).^54,55^ The MarvinSketch software was used to draw the 2D chemical structures (https://chemaxon.com/products/marvin).

The scientific impact of DFT on physics, chemistry and biology is enormous. The computational efficiency of DFT means that larger (more realistic) systems can be treated, giving electronic structure theory much more predictive power and expanding its potential for applications. This trend is further boosted by continuing improvements in computer performance. Researchers worldwide use the DFT method in an intensive way, making it the most popular QM method in present use. The accuracy of DFT has increased notably over the last few decades, being quite suitable to the study of a range of chemical systems.^56^

The molecular docking was conducted with the tool AutoDock Vina (version 1.1.2),^57^ as implemented in the MolAr (Molecular Architecture) software.^58^ For the crystallographic M^pro^ and RdRp polymerase structures preparation, the loop regions were rebuilt using the Modeller.^59^ As such, the ions and water molecules were removed from the original PDB, with the exception of water molecules that were in the M^pro^ and RdRp active sites. Additionally, the polar hydrogen atoms was added in Chimera software^60^ according to the protonation state of the receptor at a pH value of 7.4. For the docking protocol, both M^pro^ and RdRp enzymes and the structures of halogenated favipiravir tautomeric forms were used as receptor and ligands, respectively. Hence, the grid box was centered on the co-crystallized ligand (6-[ethylamino]pyridine-3-carbonitrile) of SARS-CoV-2 virus M^pro^ enzyme (5R82), and the coordinates were *x* = 12.053, *y* = −0.871 and *z* = 24.157, with 1 Å spacing. As such, the same procedure was performed for the enzyme of SARS-CoV-2 RdRp polymerase, the grid box was centered on the co-crystallized ligand cytidine-5′-triphosphate (3H5Y), and the coordinates were *x* = 30 594, *y* = 0.628 and *z* = −0.780, with about of 1 Å spacing. Finally, the docked poses obtained along in this procedure were then selected on the basis of scoring functions as well as protein–ligand interactions. Binding interaction figures were generated using Discovery Studio 2017 R2.^61^ We also provide a theoretical estimation for the acute toxicity by use of LD_50_ values obtained from a rat model-based admetSAR predictor, which is freely available online at http://biosig.unimelb.edu.au/pkcsm/prediction.

## 3. Results and discussions

### 3.1 Electrostatic potential map

**Fig. 2:**
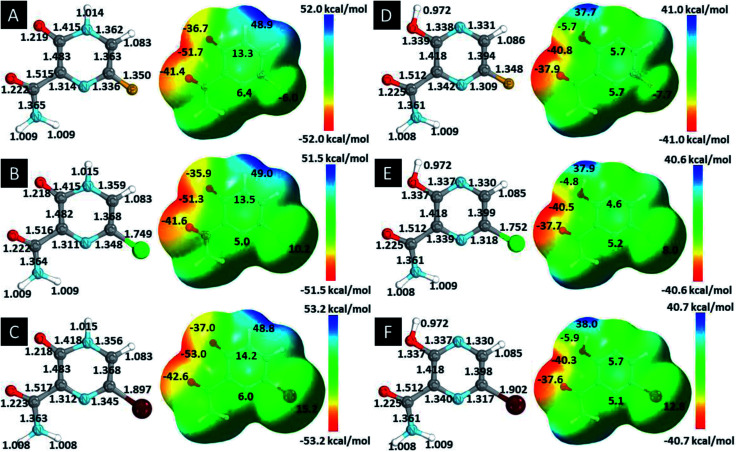
Optimized structures and ESP maps (with surface isovalue of 0.0004) for the halogenated favipiravir tautomeric forms: (a) 1-F; (b) 1-Cl; (c) 1-Br; (d) 2-F; (e) 2-Cl and (f) 2-Br.

In this *in silico* study, halogenated favipiravir tautomeric forms were firstly investigated by DFT and TDDFT calculations. From now on, the 1-F, 1-Cl and 1-Br will refer to the keto form of the derivatives. On the other hand, the 2-F, 2-Cl and 2-Br will refer to the enol form. Fig. 2(a)–(f) shows the optimized structures, bond lengths and ESP maps for each derivative. As seen in Fig. 2, the change from F to Cl and Br atoms do not affect significantly the molecular structure, in general, except for the C–F to C–Cl and C–Br bond lengths, which are longer than expected (due to their higher atomic radius). However, the changes in the tautomer structures are mainly seen in the OH group, which shows longer C–O and shorter O–H bonds, though some shifts in the N–C and C–C bond lengths at the main ring are seen as well. Hence, it is well-known that such structural parameters are, in principle, dependents of the nature of bonded atoms and their chemical environment. Also, we observed an increase in the dipole moment with the replacement of fluoride in the favipiravir structures. Finally, all these parameters determined for the optimized structure are consistent with the literature.^62,63^

As for the ESP maps shown in Fig. 2(a)–(f), the charge distribution is oriented towards the O atoms and N–H or O–H functional groups for the derivatives. These high negatively (red) and positively (blue) charged surfaces, or respectively, electrons acceptor and donor areas, show the most favorable regions for interaction between molecules, thus having a higher reactivity.^1^ As such, the halogenic substitution does not affect the charge distribution significatively; however, the tautomer molecules are lesser polarized than the original structure due to a charge stabilization in the O–H group.

### 3.2 Tautomerization mechanism simulation

According to Fig. 1, the agent favipiravir undergoes a tautomerism process that gives rise to its tautomeric form. Hence, from a drug-metabolism perspective, this process occurs *via* a water-based proton transfer mechanism or without water-assisted. Hence, the TS obtained through DFT calculations for both cases are shown in Table 1. These calculations were carried out in gas phase and water as implicit solvent (PCM). In all cases, a single imaginary frequency was obtained as shown in Table 1, confirming the achievement of the TS.

Transition states (TS) of the tautomerism mechanism of the water-assisted process and without water, in gas phase and implicit solvent (water), respectively

**Table:** 

Gas phase				
Cl	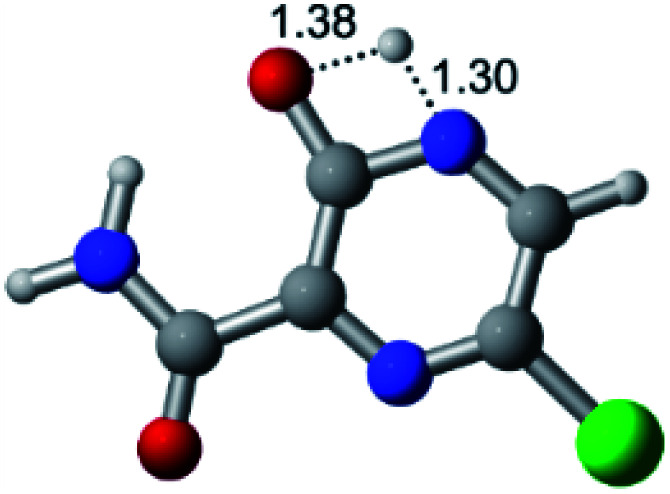	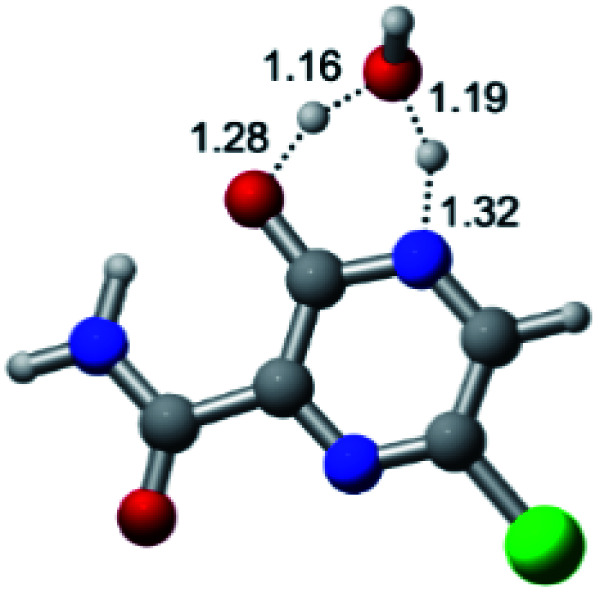	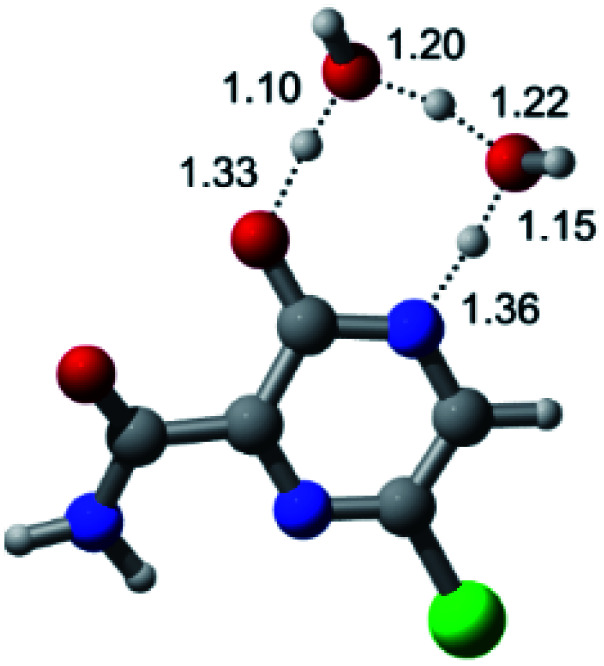	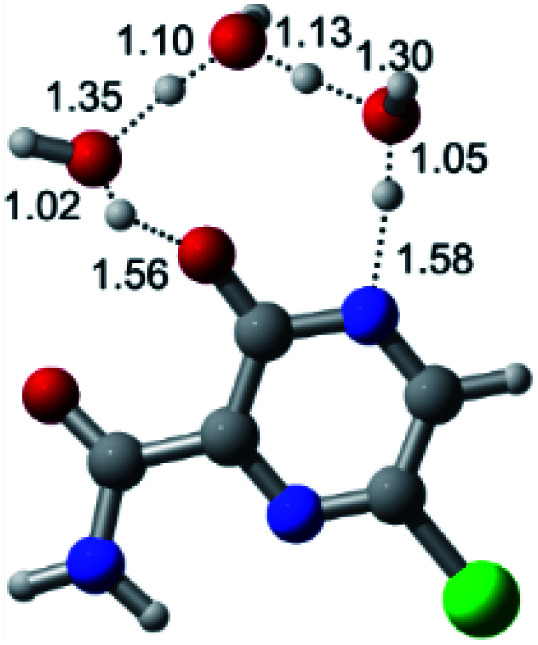
	Energy = −967.82 a.u.	Energy = −1044.31 a.u.	Energy = −1120.76 a.u.	Energy = −1197.21 a.u.
	NIMAG = −1878.14 cm^−1^	NIMAG = −1453.72 cm^−1^	NIMAG = −1216.46 cm^−1^	NIMAG = −357.30 cm^−1^
Br	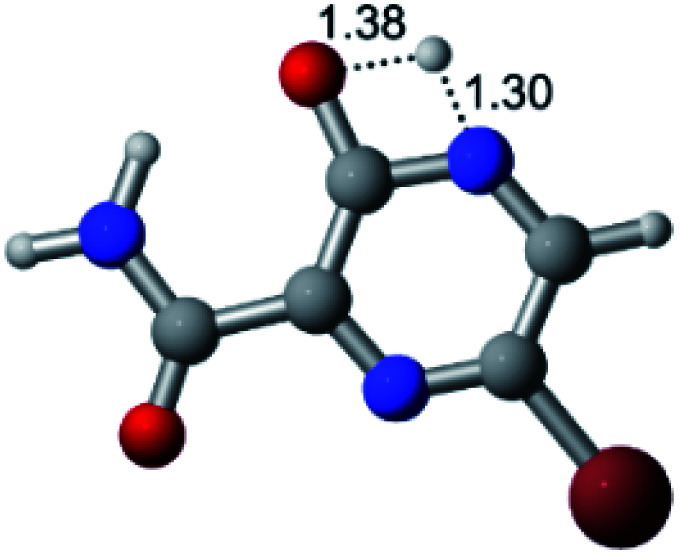	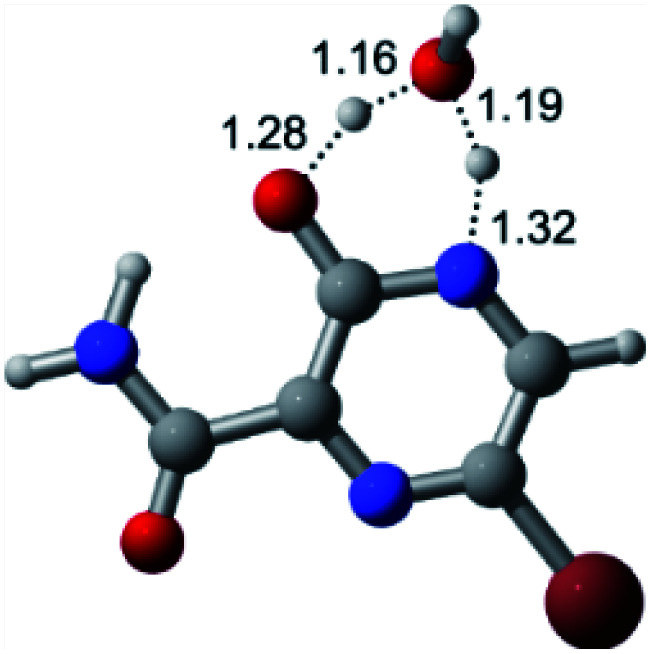	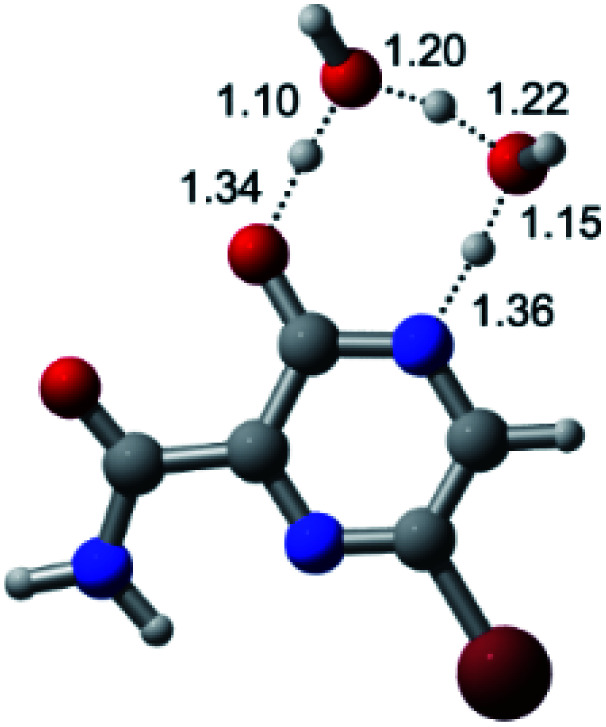	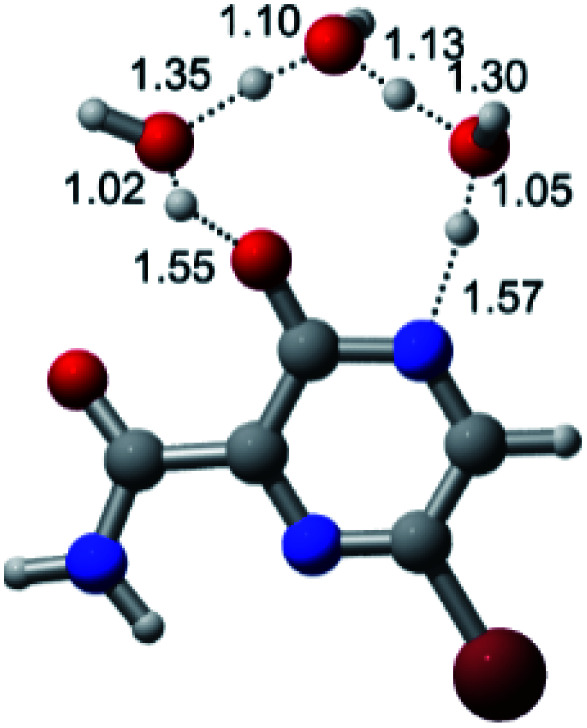
	Energy = −3079.35 a.u.	Energy = −3155.84 a.u.	Energy = −3232.29 a.u.	Energy = −3308.74 a.u.
	NIMAG = −1877.99 cm^−1^	NIMAG = −1450.93 cm^−1^	NIMAG = −1214.20 cm^−1^	NIMAG = −355.42 cm^−1^
F	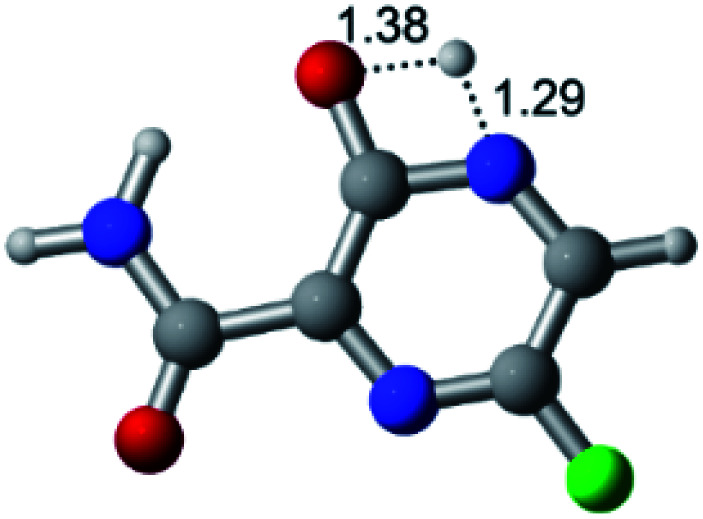	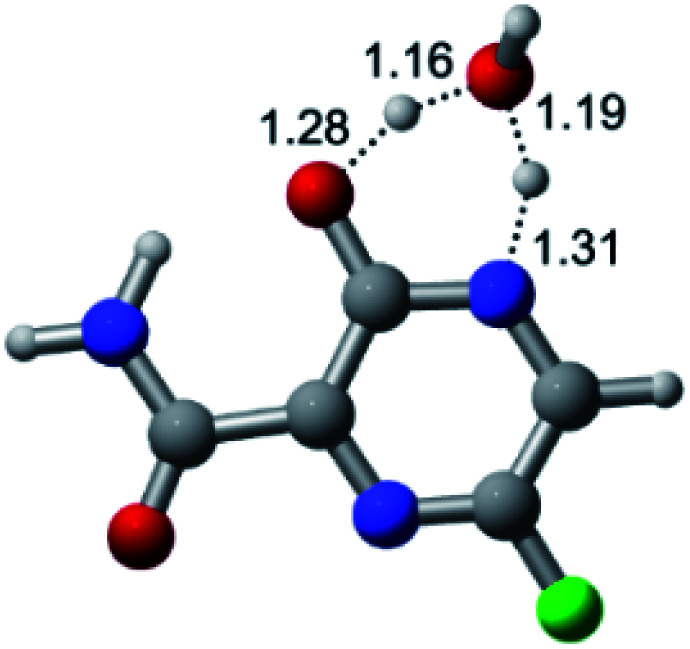	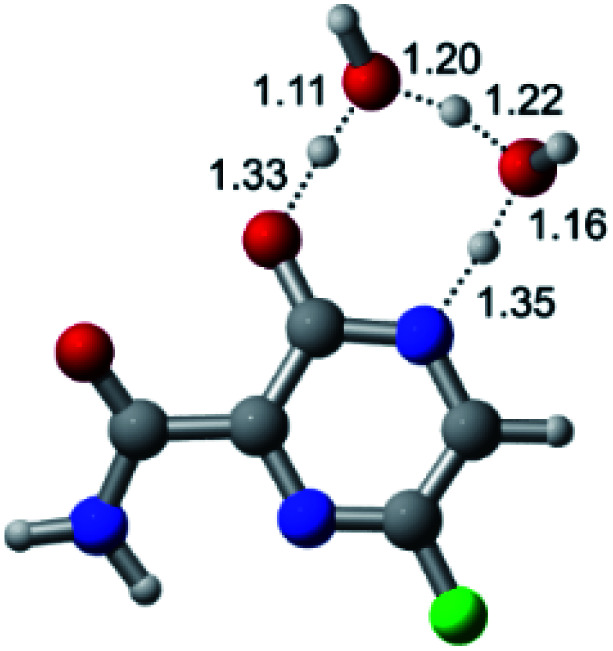	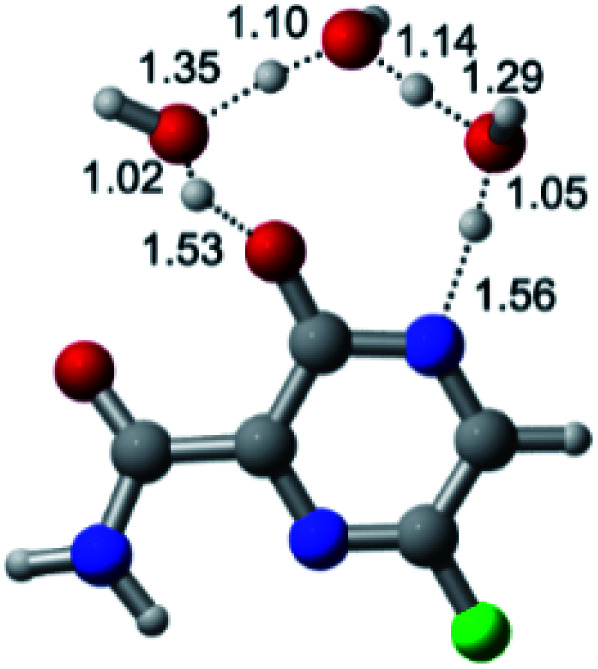
	Energy = −607.47 a.u.	Energy = −683.96 a.u.	Energy = −760.41 a.u.	Energy = −836.86 a.u.
	NIMAG = −1915.94 cm^−1^	NIMAG = −1003.52 cm^−1^	NIMAG = −1249.76 cm^−1^	NIMAG = −417.01 cm^−1^

**Table:** 

Implicit solvent (water)				
Cl	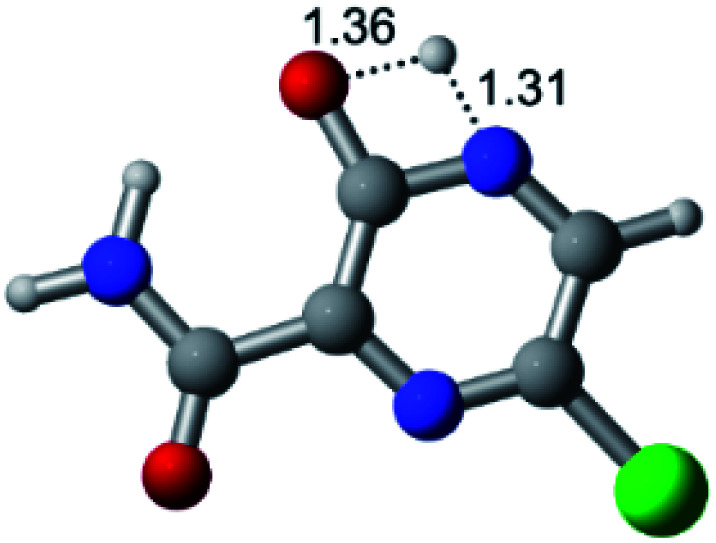	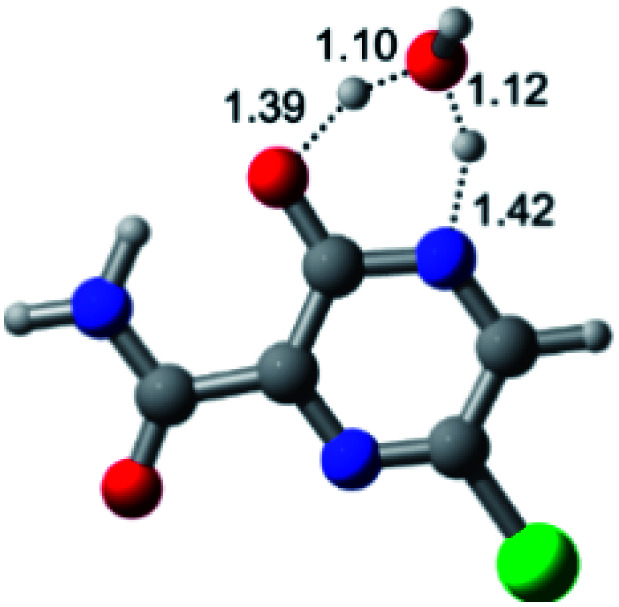	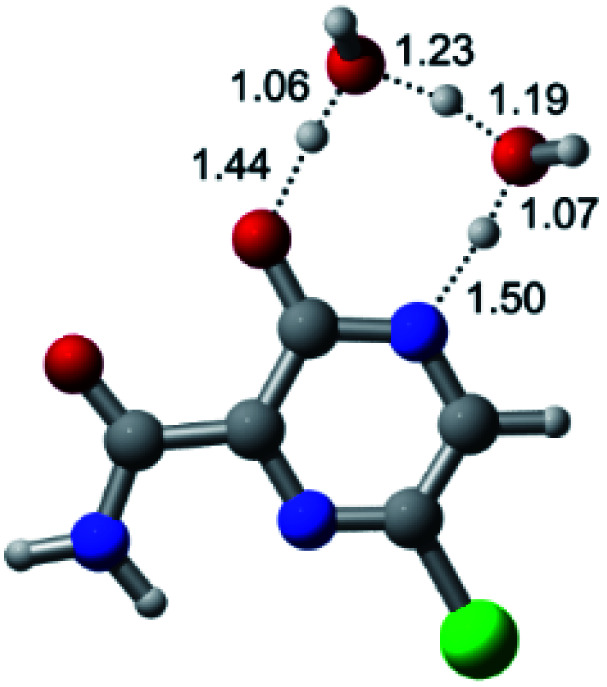	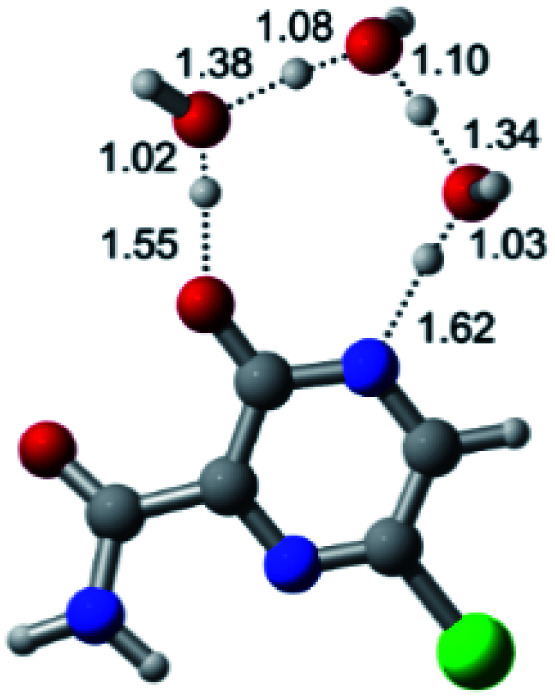
	Energy = −967.84 a.u.	Energy = −1044.33 a.u.	Energy = −1120.79 a.u.	Energy = −1197.24 a.u.
	NIMAG = −1913.03 cm^−1^	NIMAG = −941.77 cm^−1^	NIMAG = −738.36 cm^−1^	NIMAG = −101.61 cm^−1^
Br	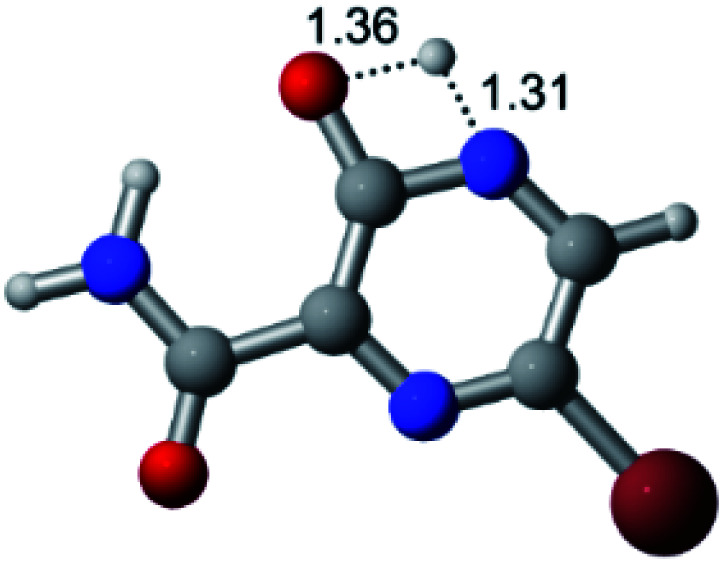	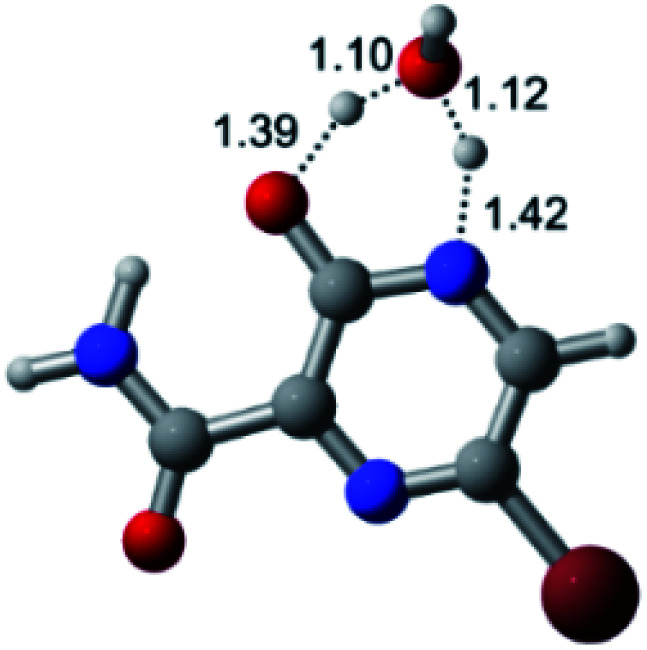	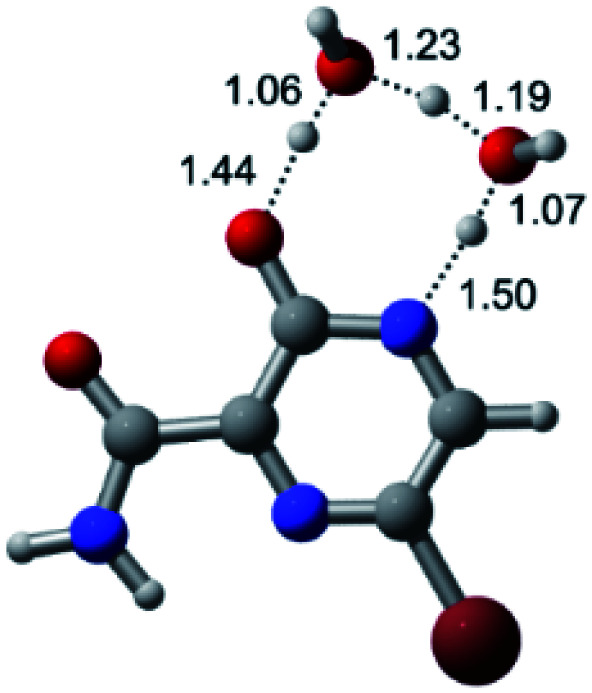	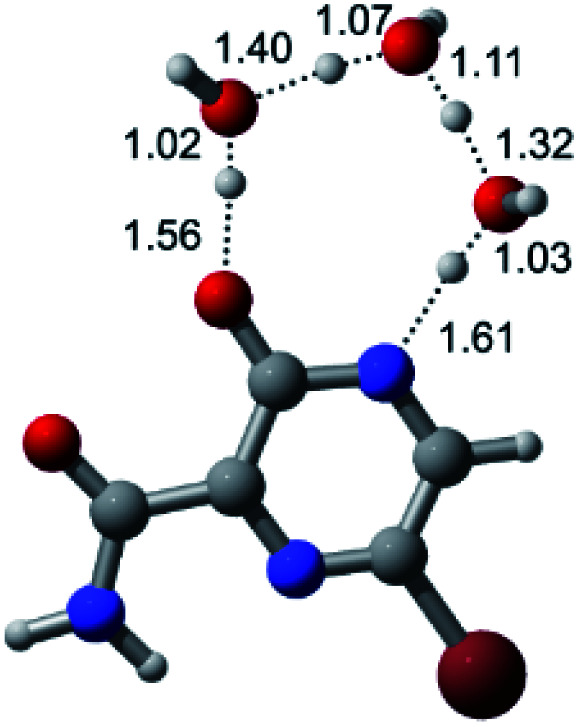
	Energy = −3079.37 a.u.	Energy = −3155.86 a.u.	Energy = −3232.32 a.u.	Energy = −3308.77 a.u.
	NIMAG = −1913.72 cm^−1^	NIMAG = −930.60 cm^−1^	NIMAG = −744.43 cm^−1^	NIMAG = −133.15 cm^−1^
F	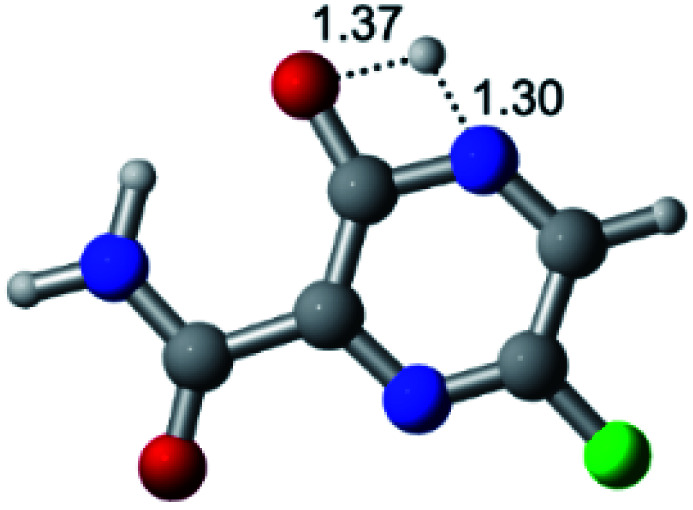	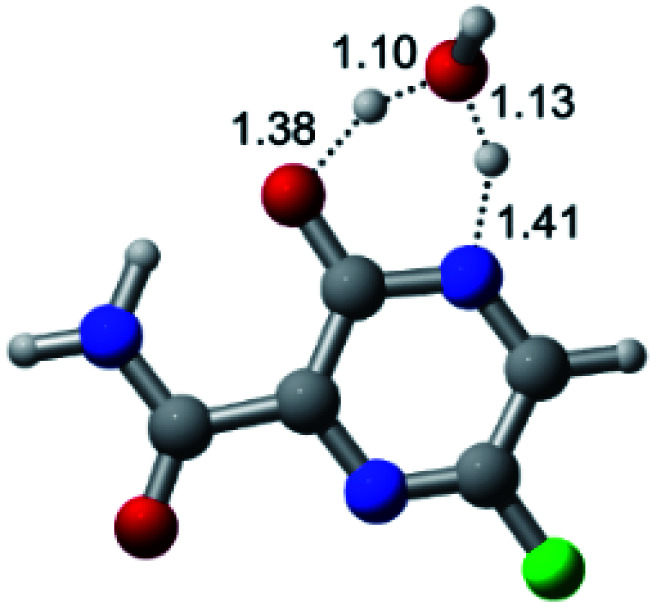	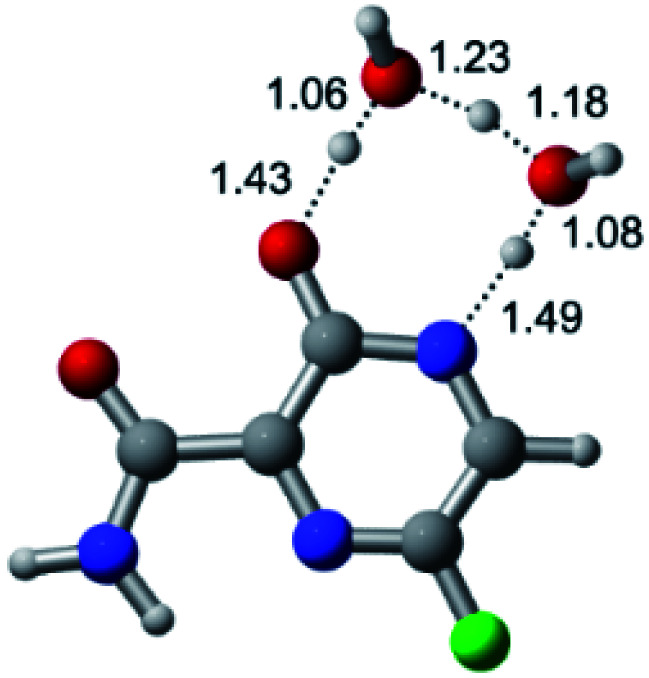	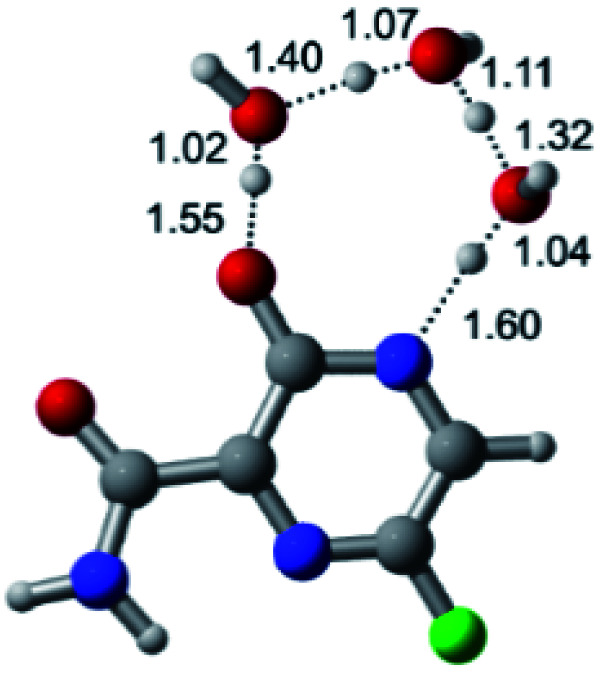
	Energy = −607.48 a.u.	Energy = −683.98 a.u.	Energy = −760.44 a.u.	Energy = −836.89 a.u.
	NIMAG = −1915.94 cm^−1^	NIMAG = −1003.52 cm^−1^	NIMAG = −753.77 cm^−1^	NIMAG = −139.32 cm^−1^

According to Table 1, the tautomeric mechanism without water molecules showed the highest TS energy, this is, less stabilizing energies. On the other hand, the water-assisted proton transfer mechanism led to more stabilizing TS geometries and decreased energies. We also can observe that by increasing the number of water molecules in the tautomerism process, it is possible to obtain more stable geometries for the TS structure, making this process energetically more favorable. Another important trend that should be highlighted is the fact of increasing the electronegative character of the atom directly bound to the ring (Br < Cl < F). Our findings show that the replacement of the chlorine atom from favipiravir with more electronegative atoms, such as fluorine, this leads to higher TS energy values. On the other hand, in case chlorine is replaced by a less electronegative atom, such as bromine, this feature leads to decreased TS energy values. According to these results, we can observe that there were no significant differences in energy for solvent calculations. However, structural changes can be noticed by the variation of distances among atoms.

### 3.3 Spectroscopic and spectrometric properties

**Fig. 3:**
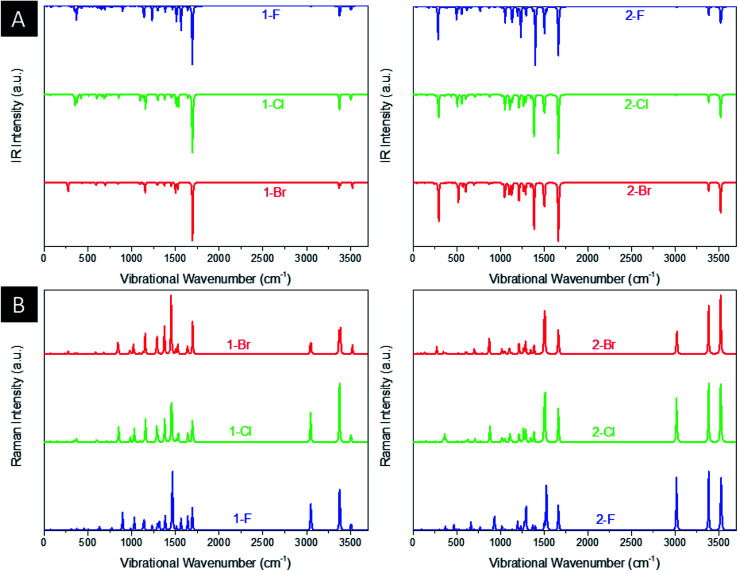
Computed (a) IR and (b) Raman spectra of the derivatives.

Fig. 3(a) and (b) shows the infrared (IR) and Raman spectrum of title compounds. As is well known, the DFT calculations usually overestimate the values of energy and consequently frequency. Thus, to overcome the high vibrational wavenumbers, all frequency-derived spectra were scaled with a factor of 0.940 in relation to the experimental work of Sebastian *et al.* (2016) with 5-chloro-*N*-(3-nitrophenyl)pyrazine-2-carboxamide.^64^ As a result, the 1-F derivatives present a main intense peak at 1694 cm^−1^ related to the stretching of C=O groups and an important signal at 1233 cm^−1^ assigned to the stretching of C–F, being blueshifted to 1100 cm^−1^ and 1091 cm^−1^ for Cl and Br, respectively. The peaks localized around 3375 cm^−1^ and 3508 cm^−1^ are related to, respectively, the symmetrical and asymmetrical stretchings of NH_2_ and NH groups. As for the intense Raman signal at 1464 cm^−1^, it is characterized by the deformation of the aromatic ring by asymmetrical stretchings of N ring atoms. All peaks discussed for these structures are both IR and Raman active-modes, showing signals located at the same IR and Raman wavenumbers. The discussed signals are shifted from the 1-F molecules, thus the C=O stretching peak is blueshifted to 1664 cm^−1^ and the C–F stretching to 1196 cm^−1^, however, the Cl and Br heteroatoms are redshifted to 1108 cm^−1^ and 1102 cm^−1^, respectively. For the tautomers, it is noticed the appearance of significant peaks at 1400 cm^−1^ and 3529 cm^−1^, associated, respectively, to C–O and O–H stretchings of OH group. It is worth notice that 2-F molecules present an intense IR active-mode around 310 cm^−1^, in which is related to the bending of NH_2_ group. In general, the estimated wavenumbers are in agreement with other theoretical works^38,63^ and the experimental report for 5-chloro-*N*-(3-nitrophenyl)pyrazine-2-carboxamide,^64^ as well.

**Fig. 4:**
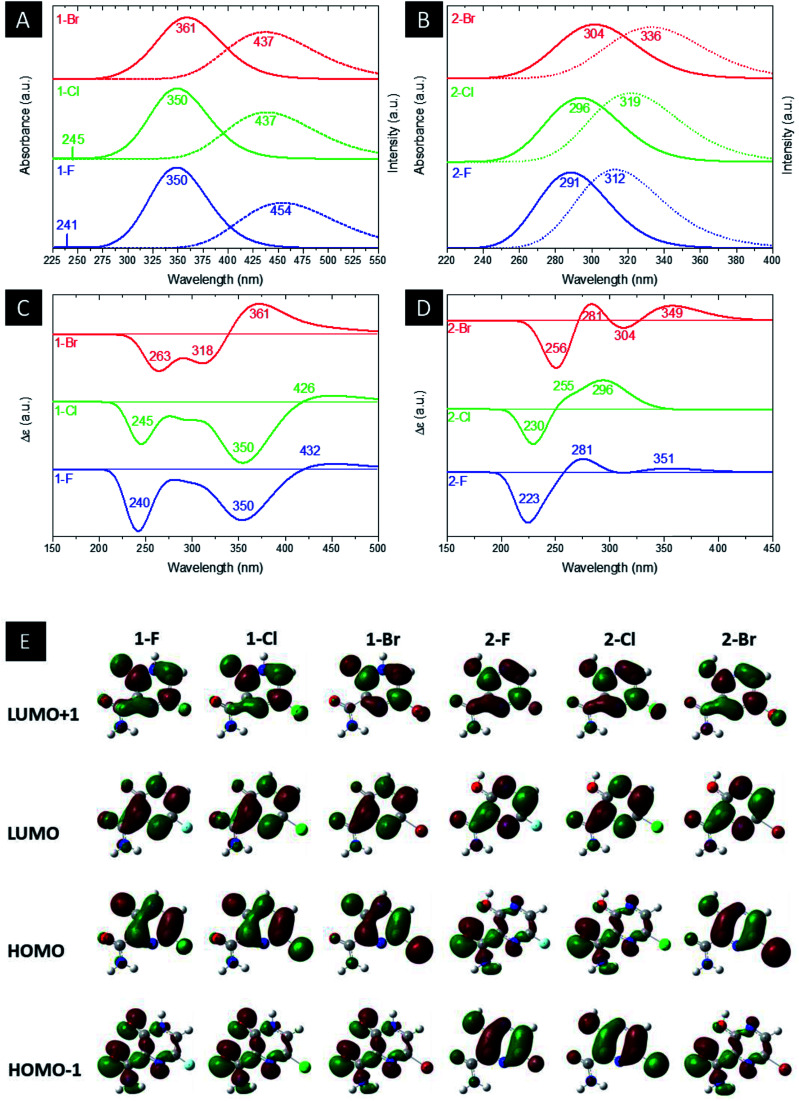
Computed (a and b) UV-Vis and emission, (c and d) ECD spectra and (e) MOs shapes (with a surface isovalue of 0.03) of both (a) 1 and (b) 2 derivative tautomer forms.

Fig. 4(a) and (b) illustrate the computed UV-Vis absorption and emission spectrum for the halogenated favipiravir compounds. For the UV-Vis absorption spectrum of 1-F and 1-Cl derivatives, it is found an intense band at 350 nm and a very weak signal of high energy transition at 241 or 245 nm, respectively. In contrast, the 1-Br molecule shows only a small redshifted for the most intense band, which is located at about 361 nm (see Fig. 4(a)). Note that the dotted line in Fig. 4(a) and (b) corresponds to the emission spectra for such compounds. From the emission and UV-Vis absorption spectra of these compounds were calculated the Stokes shifts ranging from 76 nm (1-Br) to 104 nm (1-F). As it is shown, the lower energy part of the absorbance bands is overlapped with the higher energy part of the emission bands, forming a sensitive Förster Resonance Energy Transfer (FRET) region from intramolecular mechanisms.^65^ As for the emission bands, it can be noted that a large peak at about 437 nm for both 1-Cl and 1-Br compounds. In contrast, we can observe a redshift in computed emission spectra for the 1-F compound, which have emission wavelengths at 454 nm. On the other hand, as shown in Fig. 4(b), the 2-F derivatives present only an intense band in the UV-Vis absorption and emission spectra, with major blueshifts in the band position, ranging from 291 nm to 304 nm associated respectively to 2-F and 2-Br structures. The computed excitonic transitions, their related orbitals and the Stokes shifts for the halogenated favipiravir tautomeric forms are organized in Table 2.

**Table:** Analysis of UV-Vis and emission spectra of both 1 and 2 derivative tautomer forms

Molecule	Excitonic transition	Absorbance wavelength (nm)	Emission	Emission wavelength (nm)	Stokes shift (nm)
1-F	HOMO → LUMO	350	LUMO → HOMO	454	104
	HOMO−4 → LUMO	241			
1-Cl	HOMO → LUMO	350	LUMO → HOMO	437	87
	HOMO−4 → LUMO	245			
1-Br	HOMO → LUMO	361	LUMO → HOMO	437	76
2-F	HOMO−1 → LUMO	291	LUMO → HOMO	312	21
2-Cl	HOMO−1 → LUMO	296	LUMO → HOMO	319	23
2-Br	HOMO−2 → LUMO	304	LUMO → HOMO	336	32

From Table 2, it is found that the excitonic transitions are mainly HOMO → LUMO for the 1-F type tautomers, while the 2-F type transitions happen from lower levels of the valence band to the LUMO orbital, as shown in Table 2. It is worth noticing that higher Stokes shifts of 1-F type derivatives may be a clue to a Excited States Intramolecular Proton Transfer (ESIPT).^66^ In this framework, we propose from the higher Stokes shifts that the 1-F molecule and their halogenic species are part of an ESIPT mechanism, in which the excited 1-F molecules are quickly phototautomerized into excited 2-F derivatives due to a proton transfer between the aromatic N–H and its O=C neighbor, and returning to the original structure by a reverse proton transfer after the radiative decay. However, as seen in the lower Stokes shifts, the same do not occur to the 2-F derivatives.

Computed ECD data are also shown in Fig. 4(c) and (d). In the 1-F derivatives spectra, three signals are identified in the region between 220–440 nm, two being negatives (240–263 nm and 350–361 nm) and one positive (361–432 nm). As such, the 2-F and 2-Cl derivatives show three signals in the region of 200–360 nm, one negative (223–230 nm) and two positives (255–281 nm and 296–351 nm); however, there are four observed signals around 225–450 nm for the 2-Br tautomer, two being negatives (256 nm and 304 nm) and two positives (281 nm and 349 nm). Thus, it is analyzed that the change of heteroatom causes shifts in the positions and intensities of ECD signals, being the most significant modifications related to Br substitutions. Fig. 4(e) shows the LUMO+1, LUMO, HOMO and HOMO−1 shapes. From the HOMO and LUMO orbitals energies, the HOMO–LUMO gap and other electronic properties of the derivatives in their gas and water phase are calculated and organized in Table 3. Therefore, there are patterns in the HOMO–LUMO gaps, in which Cl derivatives have slightly higher energy and Br lower energy in comparison to their 1-F and 2-F tautomers. The HOMO–LUMO gaps are in accordance to the ESP maps as seen in Fig. 2, which 1-F derivatives are more chemically reactive than 2-F due to their significantly smaller energy gaps. Along the energy gaps, other electronic properties are slightly shifted as well.

**Table:** Electronic properties of the derivatives in gas phase and solution

Molecule	HOMO–LUMO (eV)	Hardness (eV)	Softness (eV)	Mulliken electronegativity (eV)	Electrophilicity (eV)
**Gas phase**					
1-F	3.88	1.94	0.51	−5.02	50.40
1-Cl	3.91	1.95	0.51	−4.98	49.70
1-Br	3.83	1.91	0.52	−4.98	49.70
2-F	4.62	2.31	0.43	−5.02	50.40
2-Cl	4.64	2.32	0.43	−5.00	50.00
2-Br	4.58	2.29	0.44	−4.96	49.20

**Solution**					
1-F	3.95	1.97	0.51	−4.87	47.53
1-Cl	3.98	1.99	0.50	−4.84	46.85
1-Br	3.89	1.94	0.51	−4.83	46.75
2-F	4.70	2.35	0.42	−4.96	49.2
2-Cl	4.67	2.33	0.43	−4.91	48.31
2-Br	4.59	2.29	0.44	−4.87	47.53

**Fig. 5:**
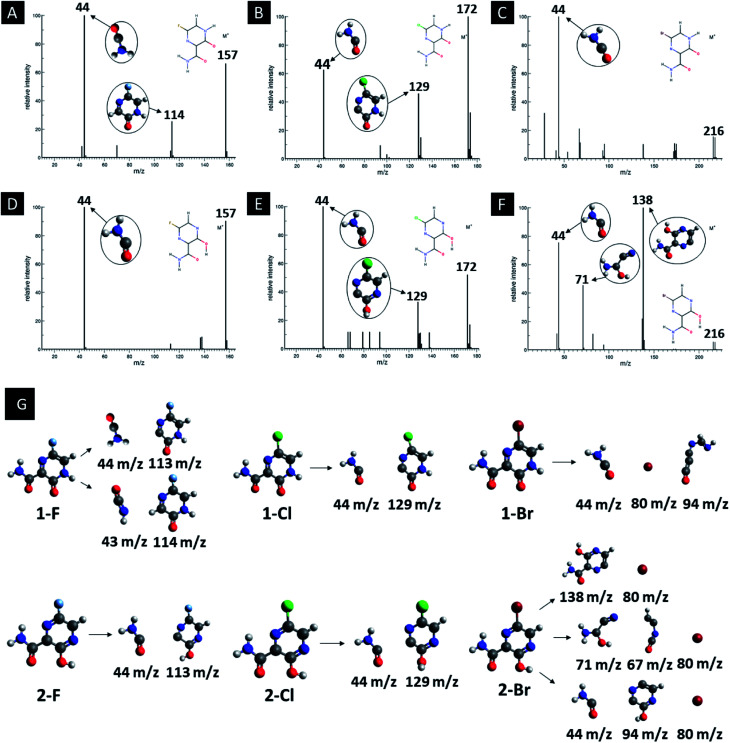
EI-MS diagrams, trajectories and intermediaries of all studied derivatives. (A) 1-F, (B) 1-Cl, (C) 1-Br, (D) 2-F, (E) 2-Cl and (F) 2-Br structures.

In addition, computed EI-MS spectrum were done as a mean to identify and differentiate each derivative and as well to understand their intermediary structures. Fig. 5 shows the EI-MS spectrum and their respective trajectories, as well as intermediaries for all studied compounds. In every diagram is observed a peak at 44 *m*/*z* related to the linear group fragmentation outside the main ring of their source molecules. It is observed a pattern in the fragmentation of both F and Cl derivatives, in which the main structures are divided into two fragments, giving rise to signals related to a linear part (44 *m*/*z*) and a cyclic part with the heteroatom (113–129 *m*/*z*). However, it is observed a third peak at 80 *m*/*z* associated to Br atoms in the Br derivatives, as seen in Fig. 5.

In order to best characterize the tautomers and understand their electronic structure, efforts have been made to obtain the NMR shieldings of the derivatives, as listing in Table 4. As such, the first is that N atoms next to the halogenic heteroatoms, and those close to OH group in the case the case of 2-F derivatives, present a relative lower shielding in comparison to other N atoms. Second, as the halogenic nuclei radius increases, the NMR shielding increases as well, ranging from 301.55 (F) to 2213.26 ppm (Br). The last conclusion to be made is that several shifts in the shieldings are observed when transitioning to solvent phase, as shown in the chemical shift Δ*σ*, however, the chemical shift of O^2−^ nuclei are significantly higher than other nuclei. Hence, the main reason for these higher values is due to interaction between tautomers and water molecules from the solvent, in which hydrogen bonds are formed between HO–H molecules and C=O groups of the derivatives compounds.

**Table:** NMR shielding on nuclei of intermediaries in gas phase and in solvent water (PCM)

Molecule	Nuclei	Shielding in gas (ppm)	Shielding in water (ppm)	Δ*σ* (ppm)
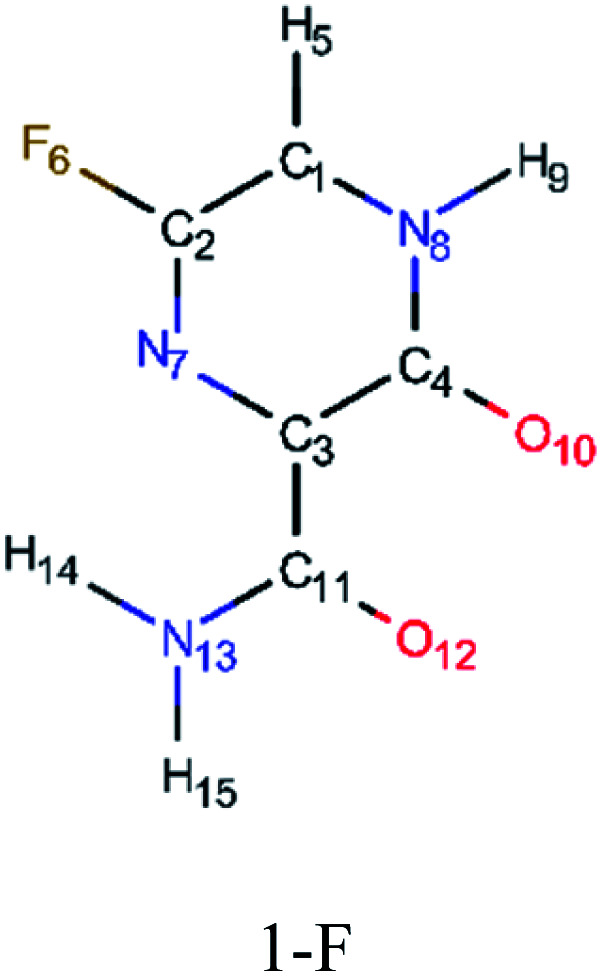	12-O	−66.26	−7.82	−58.44
	7-N	−44.26	−46.37	2.11
	10-O	−31.38	21.56	−52.94
	9-H	23.90	22.87	1.03
	5-H	24.68	24.05	0.63
	14-H	24.93	24.46	0.47
	15-H	27.08	26.61	0.47
	11-C	36.18	32.97	3.21
	4-C	48.12	44.99	3.13
	2-C	48.29	48.13	0.16
	3-C	51.00	53.71	−2.71
	1-C	79.01	73.18	5.83
	8-N	85.17	74.69	10.48
	13-N	171.85	165.03	6.82
	6-F	301.55	303.73	−2.18
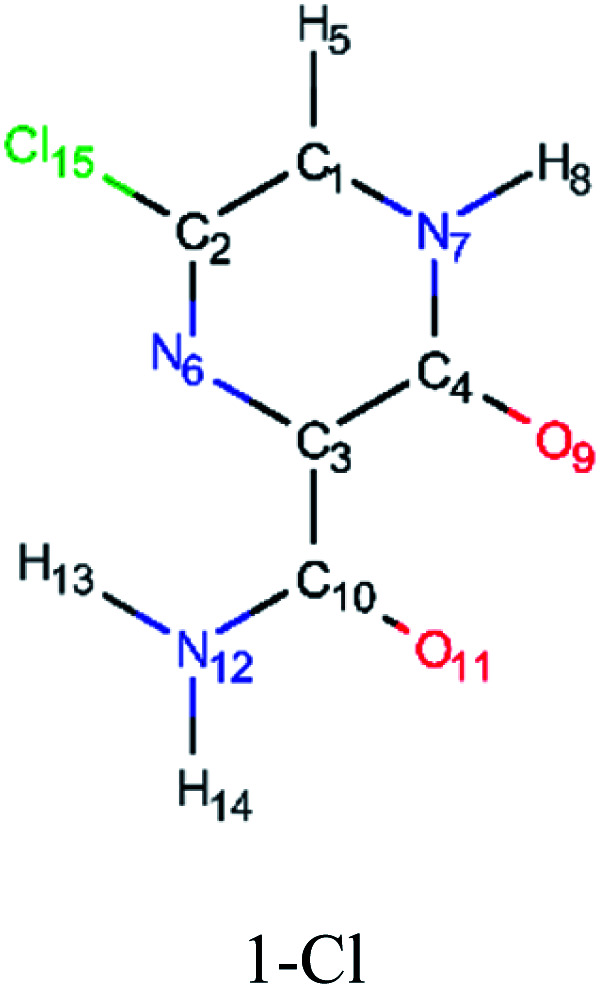	6-N	−67.44	−71.64	4.20
	11-O	−64.36	−7.16	−57.20
	9-O	−35.90	16.12	−52.02
	8-H	23.71	22.74	0.97
	5-H	24.62	24.04	0.58
	13-H	24.93	24.46	0.47
	14-H	27.10	26.64	0.46
	10-C	36.37	33.16	3.21
	4-C	48.75	45.66	3.09
	3-C	48.76	51.05	−2.29
	2-C	63.68	63.07	0.61
	1-C	66.99	61.93	5.06
	7-N	80.44	71.40	9.04
	12-N	172.46	165.63	6.83
	15-Cl	708.05	709.13	−1.08
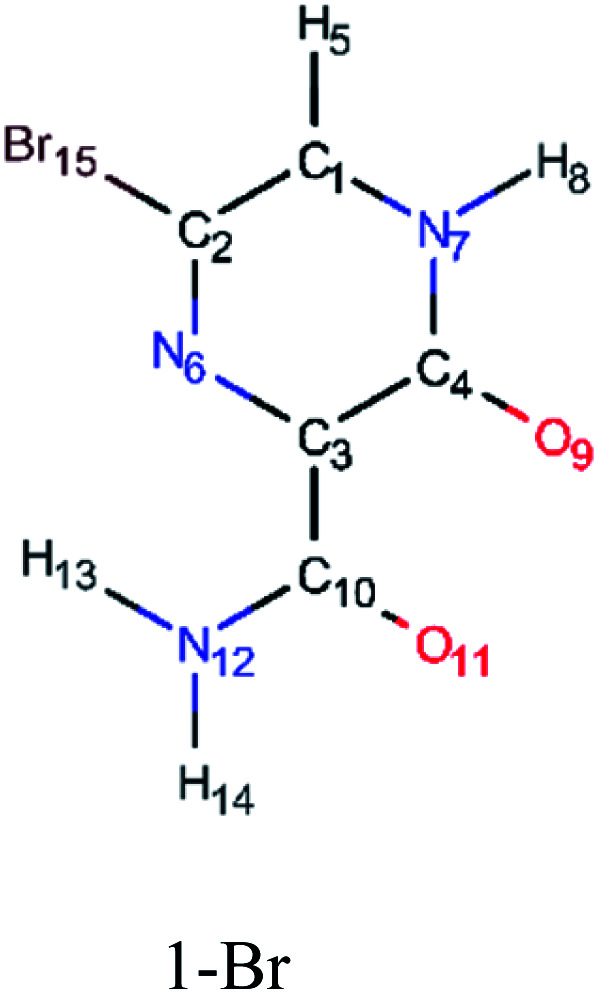	6-N	−73.15	−78.22	5.07
	11-O	−66.76	−4.09	−62.67
	9-O	−37.07	15.66	−52.73
	8-H	23.61	22.67	0.94
	5-H	24.46	23.91	0.55
	13-H	24.60	24.13	0.47
	14-H	27.06	26.61	0.45
	10-C	37.34	34.27	3.07
	4-C	49.03	45.73	3.30
	3-C	50.48	52.34	−1.86
	1-C	63.45	58.69	4.76
	2-C	65.41	64.33	1.08
	7-N	76.38	67.91	8.47
	12-N	173.11	166.53	6.58
	15-Br	2213.46	2212.032	−1998.57
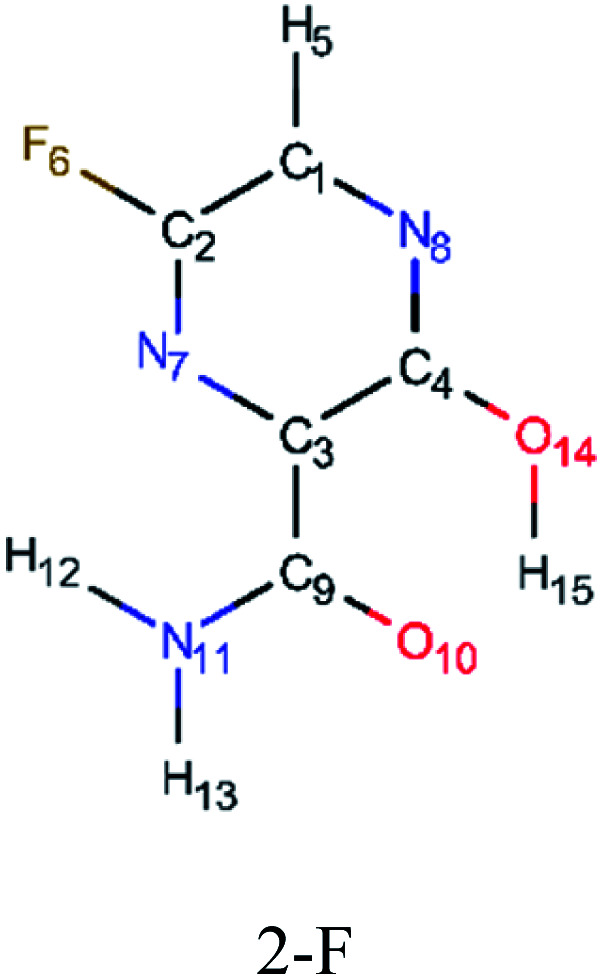	10-O	−54.27	−4.43	−49.84
	8-N	−47.51	−46.56	−0.95
	7-N	−29.68	−28.50	−1.18
	5-H	23.62	23.33	0.29
	12-H	24.39	23.98	0.41
	15-H	25.52	25.02	0.50
	13-H	26.83	26.35	0.48
	9-C	36.61	33.93	2.68
	4-C	39.07	38.86	0.21
	2-C	40.63	40.58	0.05
	1-C	62.54	59.77	2.77
	3-C	67.54	68.03	−0.49
	11-N	170.19	163.66	6.53
	14-O	190.07	196.21	−6.14
	6-F	282.12	285.63	−3.51
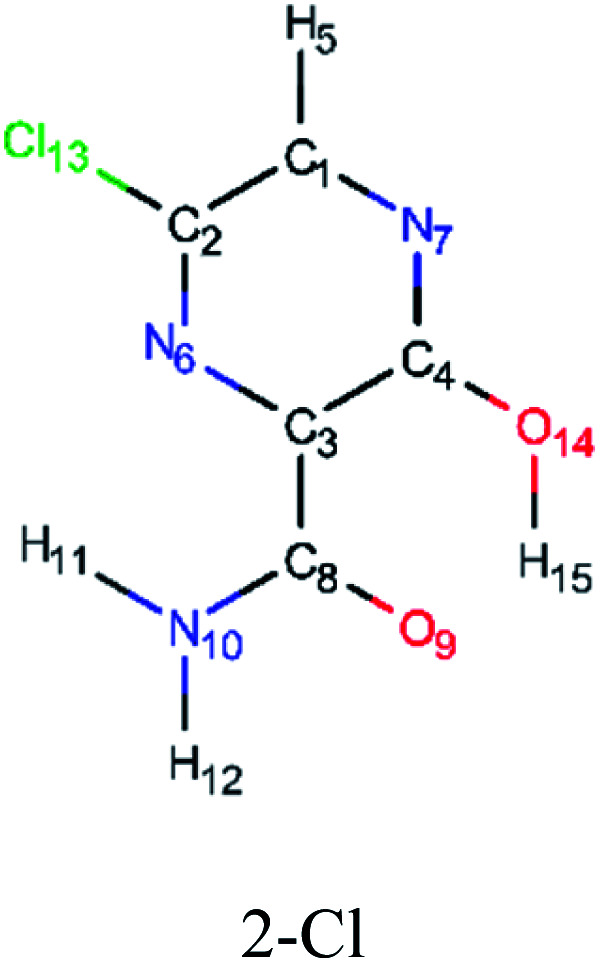	6-N	−61.30	−60.84	−0.46
	9-O	−52.74	−3.71	−49.03
	7-N	−41.79	−40.70	−1.09
	5-H	23.57	23.32	0.25
	11-H	24.31	23.91	0.40
	15-H	25.53	25.02	0.51
	12-H	26.84	26.36	0.48
	8-C	36.27	33.62	2.65
	4-C	38.52	38.23	0.29
	2-C	49.23	49.08	0.15
	1-C	51.29	49.11	2.18
	3-C	65.03	65.38	−0.35
	10-N	169.91	163.55	6.36
	14-O	189.35	195.18	−5.83
	13-Cl	686.88	689.91	−3.03
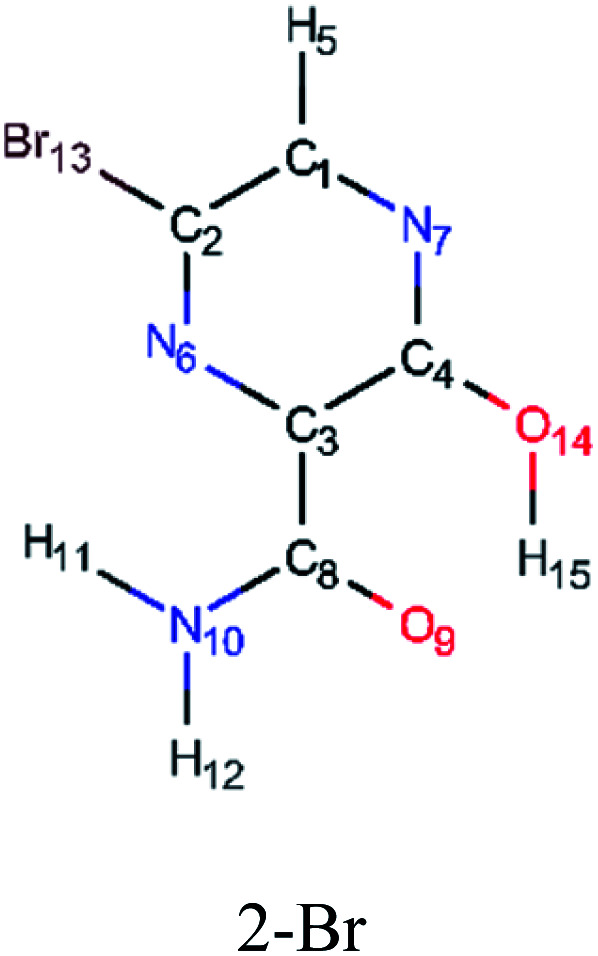	6-N	−67.78	−67.52	−0.26
	9-O	−52.47	−3.52	−48.95
	7-N	−41.34	−40.11	−1.23
	5-H	23.43	23.20	0.23
	11-H	24.30	23.89	0.41
	15-H	25.56	25.04	0.52
	12-H	26.83	26.36	0.47
	8-C	36.39	33.75	2.64
	4-C	37.97	37.70	0.27
	2-C	48.40	48.12	0.28
	1-C	48.60	46.62	1.98
	3-C	63.70	64.03	−0.33
	10-N	170.51	164.07	6.44
	14-O	189.30	195.14	−5.84
	13-Br	2182.22	2185.65	−3.43

### 3.4 Molecular docking simulation

**Fig. 6:**
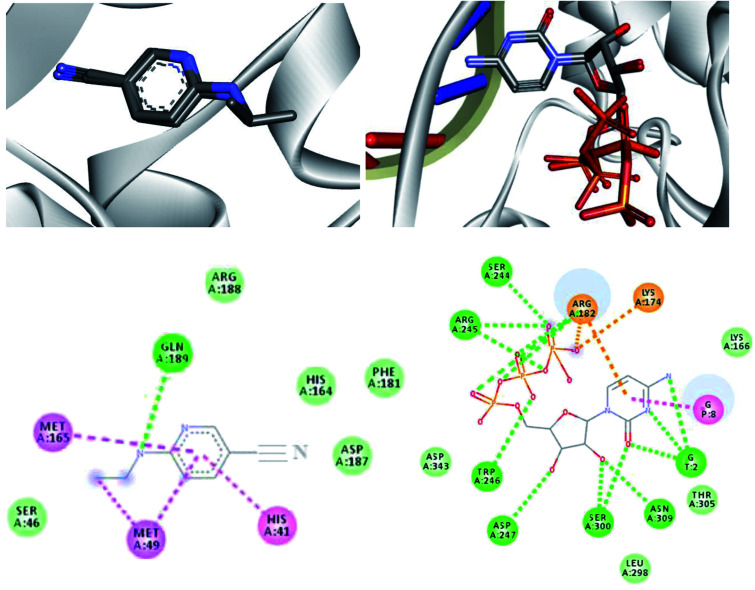
Re-docking overlaps and representation of the interactions performed by co-crystallized 6-(ethylamino)pyridine-3-carbonitrile for SARS-CoV-2 M^pro^ and cytidine-5′-triphosphate for SARS-CoV-2 RdRp sites. Interactions: green = Hydrogen bond, pink = hydrophobic and orange = coulombians.

In order to analyze the interaction modes that our drug candidates performed with SARS-CoV-2, the crystal structures of the viral M^pro^ in complex with 6-(ethylamino) pyridine-3-carbonitrile and RdRp polymerase in complex with cytidine-5′-triphosphate were downloaded from Protein Data Bank (PDB), codes 5R82 and 3H5Y, respectively.^67,68^ As the enzymes were prepared, the molecular docking protocol was started. Thus, to evaluate the ability of the algorithm to predict possible ligand orientations, re-docking calculations were performed using the MolAr software,^58^ with the implementation of the AutoDock Vina program.^57^ As such, the values extracted from RMSD (5R82 = 0.94 Å/3H5Y = 1.55 Å) indicated that Vina was able to predict the conformation that the co-crystallized ligands adopted experimentally within the SARS-CoV-2 M^pro^ active site and SARS-CoV-2 RdRp polymerase. Thus, the re-docking overlaps are shown in Fig. 6. All computed interaction energy results are shown in Table 5.

**Table:** Intermolecular interaction energies obtained through Vina

Compounds	Intermolecular interactions and energy (kcal mol^−1^)					
	RdRp (3H5Y)	H-Bond	RdRp (Y176C)	H-Bond	M^pro^ (5R82)	H-Bond
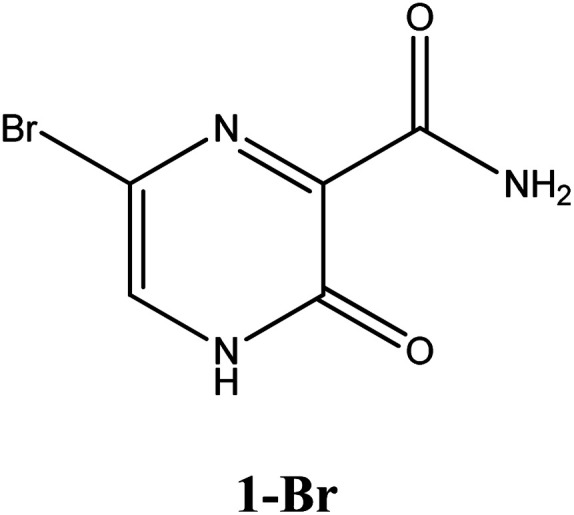	−6.5	Ser300, Asp247, Asp343, Tpr246, Asn309, G8	−5.6	Asn309, Trp246	−4.4	His41
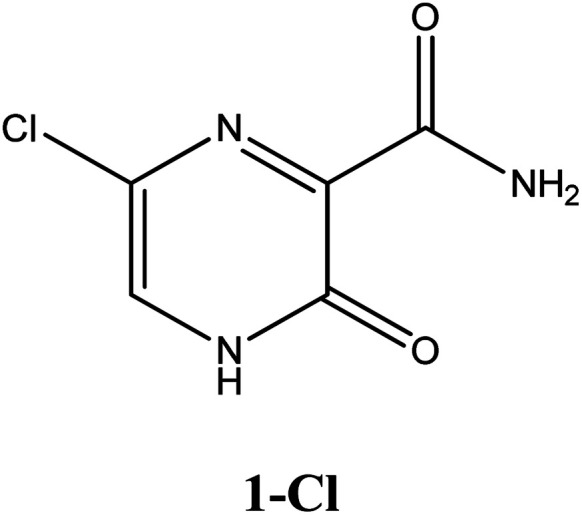	−6.5	Ser300, Asp247, Asp343, Asn309, G8	−5.6	Trp246, Asn309	−4.4	Gln189, His41
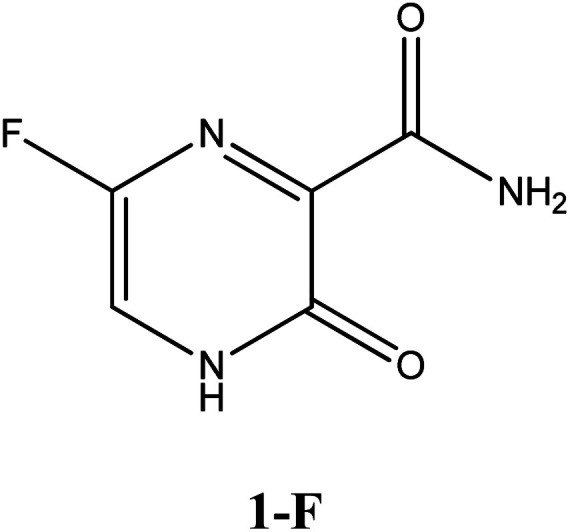	−6.4	Arg182, Asn309, Asp343, Trp246, G8	−5.5	Trp246, Asn309	−4.8	His164, Arg188
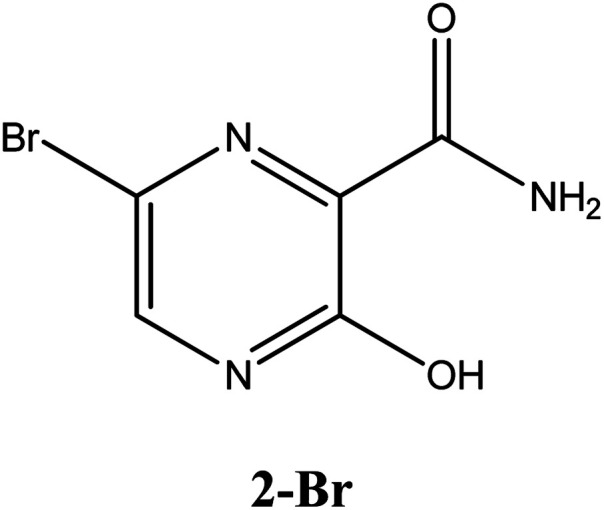	−6.3	Arg182, Asp343, G8	−5.4	Arg182, Asp247, Gln66, Ser246,	−4.6	Arg188, Gln189, His41, His164
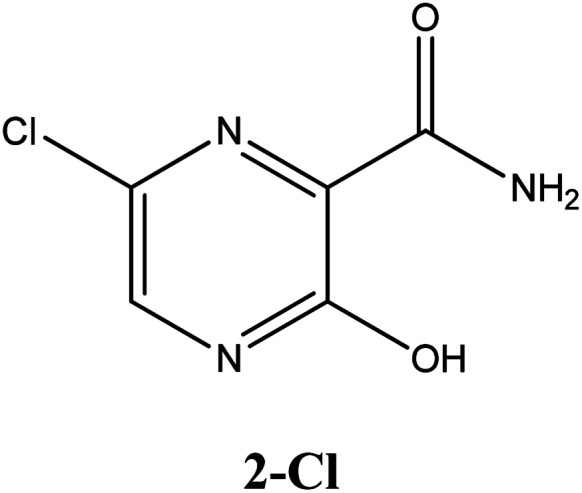	−6.3	Arg182, Asn309, Asp343, G8	−5.5	Tyr243, Asp343, Arg182, Asp247	−4.6	Gln189, His41
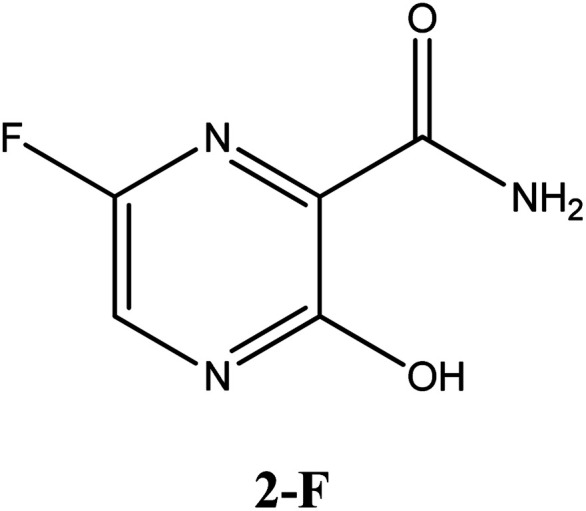	−6.3	Arg182, Asp343, Asn309, G8, Trp246	−5.4	Arg182, Asp242, Tyr243, Asp343, Asp247	−4.7	Gln189, His164, His41

According to Table 5, all drug candidates studied (*i.e.*, favipiravir and its derivatives in both tautomeric forms) interacted well with the SARS-CoV-2 RdRp site, with interaction energy values in the range of −6.3 to −6.5 kcal mol^−1^, respectively. These studied compounds showed lower interaction energy values than the co-crystallized ligand (−3.5 kcal mol^−1^) within the SARS-CoV-2 RdRp site, indicating that these compounds are very promising for the inhibition of this molecular target. Regarding the M^pro^ enzyme, the studied compounds showed interaction energy values in a range of −4.4 to −4.8 kcal mol^−1^, respectively. However, these values were not lower than the co-crystallized ligand (−9.2 kcal mol^−1^). In general, it is noteworthy that the studied compounds had a good affinity within the active site of the molecular targets, but this class of compounds interacts better with the enzyme SARS-CoV-2 RdRp site.

Regarding interactions in the SARS-CoV-2 RdRp, the 1-Br and 1-Cl compounds showed the same interaction energy value, −6.5 kcal mol^−1^, being more stable than the other compounds, that is, they have settled very well in their place. Particularly, they performed hydrogen bonding interactions with Ser300, Asp247, Asp343, Trp246, Asn309, and G8, as well as Coulombian interactions with Arg182 and hydrophobic interactions with G2 and G8, respectively. It was also observed that these compounds interact with both the enzyme and RNA, remaining well accommodated in the 3H5Y site. These intermolecular interactions carried out by these compounds are important for the inhibition of this molecular target, and this can be corroborated by the interactions performed by the co-crystallized ligand at the 3H5Y site,^67^ as shown in Fig. 6.

**Fig. 7:**
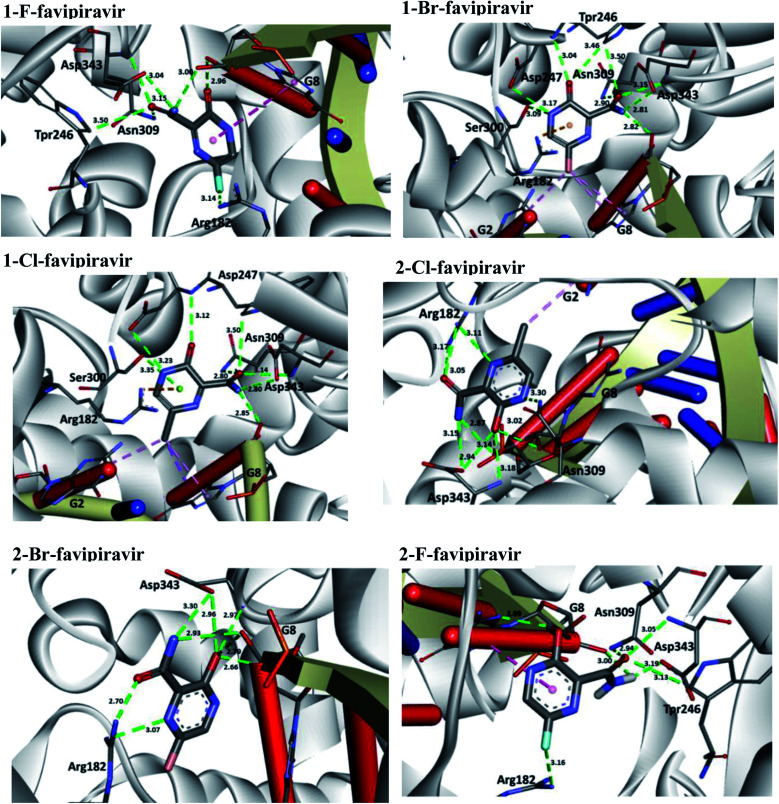
Representation of the interactions performed by favipiravir and its derivatives in the SARS-CoV-2 RNA polymerase site.

In relation to the SARS-CoV-2 RdRp site, the compounds 2-Br, 2-Cl and 2-F had the same interaction energy value (−6.3 kcal mol^−1^), performing interactions with Arg182, Trp246, Asn309, Asp343, G8 and G2 (Fig. 7). In this case, the favipiravir compound presented energy of about −6.4 kcal mol^−1^ and made interactions with Arg182, Trp246, Asn309, Asp343 and G8. A remarkable trend can be observed from these results, all tautomers (1-F, 1-Br and 1-Cl) showed better stability than their native forms (2-F, 2-Br and 2-Cl), that is, the tautomeric form of these compounds is very reactive at the SARS-CoV-2 RdRp site. Also, note that all of our drug candidates had key interactions for good affinity in the SARS-CoV-2 RdRp binding pocket, so we can suggest that favipiravir and its derivatives can effectively inhibit RNA polymerase, and in addition, being considered promising compounds for the treatment of COVID-19.^69^

Based on the promising results found by the docking of the halogenated compounds in the wild-type RdRp, we investigated the interaction modes of these ligands in a variant model of this enzyme, with mutation Y176C. According to Yashvardhini *et al.*, the mutation in which tyrosine is replaced by cysteine (Indian variant) increased the structural flexibility of the enzyme and hence can influence the viral replication.^70^ Note that the docking results for this mutant are shown in Table 5. By observing these results, we can notice that the compounds have a nice affinity in the mutant RdRp active site, with energy values ranging from of −5.4 to −5.6 kcal mol^−1^, respectively. These outcomes indicate that these ligands present a stabilizing interaction energy in both wild-type and mutant enzymes. However, our results suggest that these interaction energies were more stabilizing for the wild-type enzyme. In addition, one important trend to be highlighted consists in the fact of the compounds 1-Br and 1-Cl showed the best interaction energies in both models studied. By analysing the hydrogen bonds performed by the halogenated compounds in the mutant RdRp active site, we observe that there was a decrease of interactions for the tautomeric form (1-Br, 1-Cl, 1-F). Another trend observed is the fact of these compounds were unable to interact with RNA in the active site, *i.e.*, suggesting that this mutation is responsible for decreases the efficacy of these drug candidates.

**Fig. 8:**
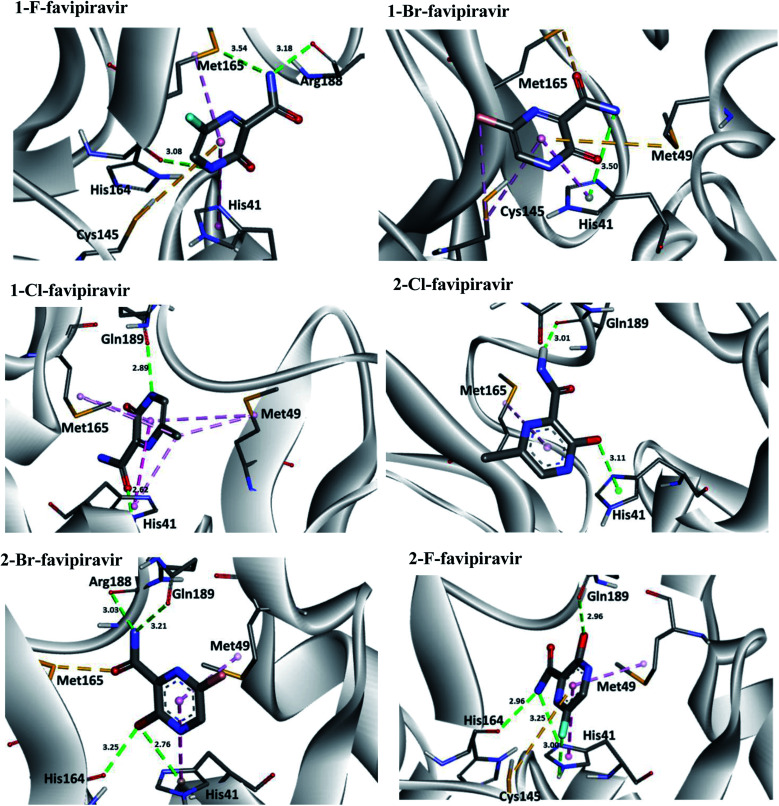
Representation of the interactions performed by favipiravir and its derivatives in the M^pro^ active site.

Regarding interactions at M^pro^, it was observed that the compound favipiravir was the one that best interacted with this enzyme, with an intermolecular interaction energy value of around −4.8 kcal mol^−1^. As such, this compound performed two hydrogen bonds with His164 and Arg188, Coulombian with Cys145 and hydrophobic interactions with Met165 and His41, as well. It is worth mentioning, according to the literature,^69,71^ that these residues are fundamental for inhibition of the viral M^pro^ (Fig. 6 and 8). In the case of the 1-Br compound, in particular, was the one that least interacted at the M^pro^ active site in relation to the other compounds. According to our results, this compound performing interactions with Cys145, His41, Met49 and His154 (see Table 5). As shown in Fig. 8, the other compounds also performed interactions with the aforementioned residues. It was observed that the tautomerism was not very significant for reactivity in this case. In general, our main objective was to determine whether the studied inhibitors could target the M^pro^ enzyme. The molecular coupling posture of each drug candidate indicated that they could, in fact, fit precisely in the substrate binding pocket.

The ADMET profile (absorption, distribution, metabolism, excretion and toxicity) was obtained, and the results for each compound are shown in Table 6.^72^ Lipinski's rule of 5 (RO5)^73^ was used to evaluate the potential of these favipiravir derivatives as orally active drugs in humans, where it establishes that a molecule to be a good drug must present values for 4 parameters multiple of 5: log *P* lower than or equal to 5, molecular mass less than or equal to 500, hydrogen bond acceptors less than or equal to 10 and binding donors hydrogen less than or equal to 5. We can note that the favipiravir derivatives violate the rules of maximum octhanol/water partition coefficient. In addition, these compounds in their native form are more toxic than their tautomers and the halogens substitution have a significantly effect on the toxicity of these studied compounds. Therefore, these compounds have considerable drug potential.

**Table:** ADMET profile of selected favipiravir derivatives^a^ (ref. ^72^)

Comp.	MW	log *P*	DH/AH	log *S*	Intestinal absorption (%)	CNS	Tox. (LD_50_)
1-F	157.10	-0.99	2/3	−1.45	86.80	−3.06	1.92
1-Cl	173.56	−0.48	2/3	−1.94	87.00	−3.07	2.12
1-Br	218.01	−0.37	2/3	−2.03	86.95	−3.06	2.13
2-F	157.10	−0.58	2/4	−1.88	86.04	−3.12	1.52
2-Cl	173.56	−0.06	2/4	−2.29	86.59	−3.11	1.8
2-Br	218.01	0.04	2/4	−2.36	86.53	−3.11	1.81

a Comp = compounds favipiravir derivatives. ADME parameters: MW = molecular weight, DH = number of H bonds donors, AB = number of H bonds acceptors, log *P* = partition coefficient, log *S* = predicted aqueous solubility, CNS = predicted central nervous system, Tox = oral rat acute toxicity (mol kg^−1^).

## 4. Conclusions

In conclusion, we have studied the electronic structure and spectroscopic properties of halogenated favipiravir tautomeric forms. These results are compatible with reported theoretical–experimental data (when available), allowing for a complete distinction in both tautomeric forms. Therefore, in this study, these effects were evaluated in order to consider favipiravir and its metabolic derivatives, since this compound has recently been used for the treatment of COVID-19. All halogenated favipiravir tautomeric forms were investigated against the SARS-CoV-2 using both M^pro^ and RdRp sites as model systems. Since all molecules have shown RNA-inhibiting properties, generating potential candidates for the COVID-19 treatment. Hence, we strongly recommend that future *in silico* studies address both biological targets, what could certainly contribute for the development of new therapies based on the combined use of drugs. The docking results showed that for wild-type and variant RdRp, the keto form was better stabilized in active site. For the viral M^pro^, these ligands presented a less stabilizing interaction in comparison with RdRp. In conclusion, we observe that the tautomeric form is indicated as nice inhibitors of the viral RdRp.

## Author contributions

Letícia Cristina Assis, Alexandre Alves de Castro and João Paulo Almirão de Jesus performed the theoretical calculations, data analysis, elaboration of initial versions of this manuscript and figures preparation; Elaine Fontes Ferreira da Cunha, Eugenie Nepovimova, Ondrej Krejcar, Kamil Kuca, Teodorico Castro Ramalho and Felipe de Almeida La Porta contributed in the technical-scientific evaluation of the final version and adjustments of language requirements.

## Conflicts of interest

There are no conflicts to declare.

## Supplementary Material
